# Tele-Rehabilitation and Tele-Diagnostics in Shoulder Disorders: Current Evidence, Challenges, and Future Directions—A Narrative Review

**DOI:** 10.3390/jcm15072694

**Published:** 2026-04-02

**Authors:** Petar Todorović, Nikola Pavlović, Andrea Kopilaš, Katarina Vukojević, Ana Čarić

**Affiliations:** 1 Department of Anatomy, Histology and Embryology, University of Split School of Medicine, 21000 Split, Croatia; petar.todorovic@mefst.hr; 2 Department of Pathophysiology, University of Split School of Medicine, 21000 Split, Croatia; nikola.pavlovic@mefst.hr; 3 Department of Family Medicine, Health Center Mostar, Bulevar Hrvatskih Branitelja b.b., 88000 Mostar, Bosnia and Herzegovina; andrea.kopilas@mef.sum.ba; 4 Department of Anatomy, School of Medicine, University of Mostar, 88000 Mostar, Bosnia and Herzegovina; ana.caric@mefst.hr; 5 Mediterranean Institute for Life Sciences, University of Split, 21000 Split, Croatia; 6 Center for Translational Research in Biomedicine, University of Split School of Medicine, 21000 Split, Croatia; 7 Medical Center Split, Poljička cesta 39, 21000 Split, Croatia; 8 Department of Radiology, University of Split School of Medicine, 21000 Split, Croatia

**Keywords:** telerehabilitation, tele-diagnostics, shoulder disorders, rotator cuff, wearable sensors, machine learning, digital health

## Abstract

**Background/Objectives**: Shoulder disorders are among the most prevalent musculoskeletal conditions, with lifetime prevalence reaching 67% and substantial associated disability and economic burden. Geographic barriers and workforce shortages impede access to optimal rehabilitation. This narrative review aims to synthesize current evidence on tele-diagnostics and tele-rehabilitation in shoulder disorders, evaluate clinical outcomes and implementation factors, and explore models for integrating these complementary approaches. **Methods**: A structured but non-systematic literature search was conducted across PubMed, Scopus, and Web of Science covering publications from January 2010 through December 2025, using terms related to telehealth, tele-rehabilitation, tele-diagnostics, and shoulder disorders. Priority was given to randomized controlled trials, systematic reviews, feasibility studies, and clinical practice guidelines in adult populations. A total of 97 articles were included in the final narrative synthesis. **Results**: Tele-diagnostic approaches demonstrate acceptable reliability for range-of-motion assessment and general diagnostic classification, though glenohumeral instability evaluation remains challenging remotely. Multiple randomized controlled trials suggest non-inferior outcomes for tele-rehabilitation compared to conventional physiotherapy across rotator cuff repair, shoulder arthroplasty, and conservative management, with generally high patient satisfaction. Certainty of evidence is currently low to moderate due to short follow-up durations, modest sample sizes, and heterogeneous protocols. Key implementation barriers include the digital divide, inability to deliver manual therapy, and insufficient long-term outcome data. **Conclusions**: Current evidence supports telehealth as a viable complement to conventional shoulder care, with the strongest evidence base for postoperative tele-rehabilitation. Hybrid care models appear clinically feasible, though widespread adoption requires standardized outcomes, longer-term trials, and strategies addressing health equity barriers.

## 1. Introduction

Shoulder disorders represent a substantial global health burden, ranking among the most prevalent musculoskeletal conditions encountered in clinical practice. Point prevalence estimates range between 7% and 26%, with lifetime prevalence reaching 67%, reflecting the chronic and recurrent nature of these conditions [1,2]. The clinical spectrum encompasses rotator cuff disorders, adhesive capsulitis, glenohumeral instability, and acromioclavicular joint disease, each requiring distinct diagnostic and therapeutic approaches [3,4]. Disability and economic consequences are considerable; shoulder pain contributes significantly to years lived with disability globally, with estimated annual per-patient costs of approximately €4139, predominantly driven by productivity losses and sick leave [1,5]. Up to 50% of patients remain symptomatic at 18 months after initial presentation, underscoring the need for effective, accessible, and sustained management strategies [4].

Traditional in-person care faces inherent access limitations that impede optimal outcomes. Geographic barriers restrict access to specialized musculoskeletal services, particularly in rural and underserved regions, where physiotherapist-to-population ratios can fall below one per 100,000 inhabitants in low- and middle-income countries [6,7]. Even in well-resourced healthcare systems, transportation costs, time away from work, and waiting periods present obstacles that are especially problematic for chronic shoulder conditions requiring prolonged supervised rehabilitation [8,9].

Telehealth, broadly defined as the delivery of healthcare services through telecommunications technology, has emerged as a promising solution to these access limitations [9]. Within this domain, two complementary applications hold particular relevance for shoulder disorders: tele-diagnostics, encompassing remote clinical assessment and diagnostic reasoning, and tele-rehabilitation, involving the delivery of therapeutic interventions at a distance [10]. Tele-diagnostic approaches utilize video-based consultations, smartphone applications, wearable sensors, and artificial intelligence to enable clinicians to evaluate patients without physical presence [11]. Tele-rehabilitation employs similar technologies to guide exercise programs, monitor progress, and provide real-time feedback, either synchronously through videoconferencing or asynchronously through digital platforms [12].

The COVID-19 pandemic served as an unprecedented catalyst for telehealth adoption across medical specialties, including musculoskeletal care [13]. Social distancing requirements and institutional restrictions on elective services compelled healthcare systems to rapidly integrate remote consultation platforms into routine clinical workflows [14]. Orthopedic and rehabilitation providers who had previously demonstrated limited engagement with telemedicine transitioned to virtual care models within weeks, generating substantial new evidence on feasibility, effectiveness, and patient acceptance [15]. This accelerated adoption revealed both the potential and limitations of remote shoulder assessment and treatment, providing a foundation for more rigorous investigation.

Emerging evidence suggests that integrating tele-diagnostics with tele-rehabilitation may offer synergistic benefits for shoulder disorder management. Remote diagnostic assessment can inform individualized rehabilitation planning, while ongoing tele-rehabilitation enables continuous monitoring and program adjustment without requiring repeated in-person visits [16]. This integrated model holds promise for extending specialized care to underserved populations, optimizing resource utilization, and potentially improving adherence through enhanced patient engagement [17]. However, questions remain regarding diagnostic accuracy, treatment effectiveness, appropriate patient selection, and implementation barriers that must be addressed before widespread adoption.

Several narrative and systematic reviews have examined telehealth in broader musculoskeletal and orthopedic contexts [9,10,18]. However, existing syntheses have predominantly addressed either tele-rehabilitation across multiple musculoskeletal conditions without shoulder-specific analysis, or telemedicine consultations in general orthopedic surgery without distinguishing diagnostic from therapeutic applications. To our knowledge, no published review has jointly examined the diagnostic and rehabilitative dimensions of telehealth specifically within shoulder disorders, nor explored the clinical and technological rationale for their integration into unified care pathways. The present narrative review addresses this gap by synthesizing shoulder-specific evidence across both domains, evaluating implementation factors, and proposing a framework for integrated tele-diagnostic and tele-rehabilitation practice. The decision to examine tele-diagnostics and tele-rehabilitation within a single review reflects both clinical and technological realities of contemporary shoulder care. Diagnostically and therapeutically, these domains are not discrete; the same wearable sensor deployed for initial range-of-motion assessment can transition seamlessly into a rehabilitation monitoring tool, and machine learning algorithms now simultaneously classify exercise performance and flag diagnostically significant movement abnormalities within a single patient interaction. Treating these functions as separable would therefore misrepresent how integrated telehealth platforms actually operate in clinical practice. Furthermore, the shoulder rehabilitation pathway inherently involves iterative reassessment throughout treatment, meaning that diagnostic and therapeutic telehealth functions naturally overlap across the care continuum. A combined review more faithfully reflects this clinical reality than two artificially separated analyses would. This narrative review aims to synthesize current evidence on the application of tele-diagnostics and tele-rehabilitation in shoulder disorders. We examine the reliability and validity of remote diagnostic approaches, evaluate clinical outcomes of tele-rehabilitation interventions for various shoulder pathologies, and discuss factors influencing patient engagement and adherence. Furthermore, we explore models for integrating these complementary approaches, identify current limitations and challenges, and propose directions for future research and clinical implementation.

## 2. Methods

This article is a narrative review conducted in accordance with the Scale for the Assessment of Narrative Review Articles (SANRA) guidelines [19]. Accordingly, a structured but non-systematic search of the literature was performed to identify key studies relevant to the scope of this review. No prospective protocol registration was undertaken, as is consistent with the narrative review format.

### 2.1. Search Strategy

**Figure 1:**
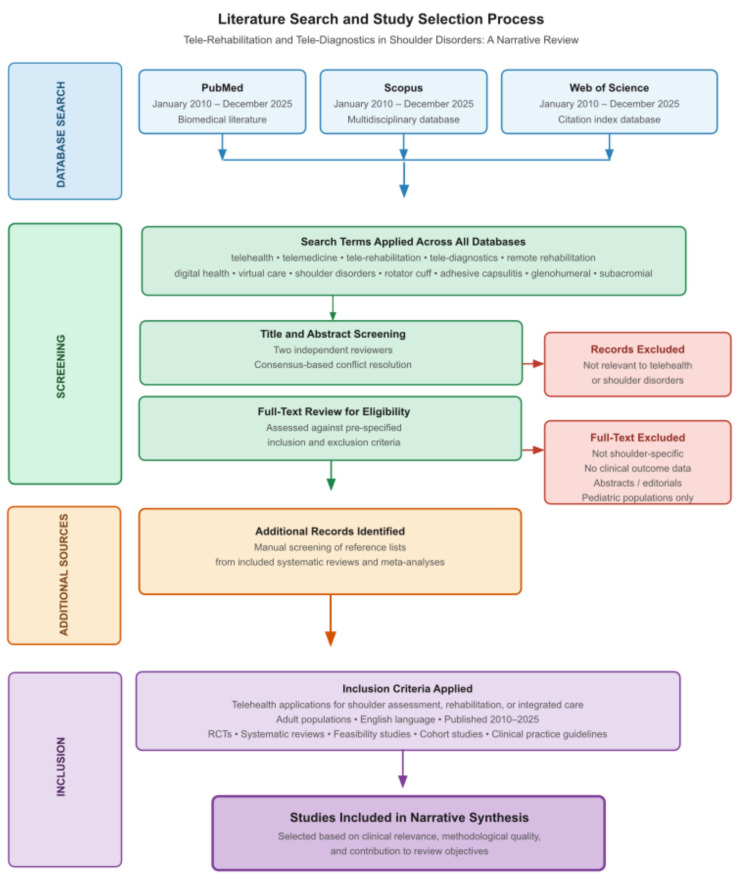
Literature search and study selection process. Schematic overview of the search and study selection strategy applied in this narrative review. Records were identified through targeted searching of three electronic databases (PubMed, Scopus, and Web of Science) covering publications from January 2010 through December 2025. Search terms were applied in various combinations across all databases. Records were screened at the title and abstract level by two independent reviewers, with consensus-based resolution of disagreements. Full-text articles were subsequently assessed for eligibility against pre-specified inclusion and exclusion criteria. Additional records were identified through manual screening of reference lists of included systematic reviews and meta-analyses. Final study selection was based on clinical relevance, methodological quality, and contribution to the review objectives. This figure represents a schematic overview of the search and selection workflow and is not intended as a PRISMA-style flow diagram; numerical counts of records identified, screened, or excluded at each stage are not reported, consistent with the narrative review methodology applied.

A targeted literature search was conducted across three electronic databases, PubMed, Scopus, and Web of Science, covering publications from January 2010 through December 2025. The following key terms were used in various combinations: telehealth, telemedicine, tele-rehabilitation, tele-diagnostics, remote rehabilitation, digital health, virtual care, shoulder disorders, rotator cuff, adhesive capsulitis, glenohumeral, subacromial, wearable sensors, machine learning, and artificial intelligence. Additional studies were identified through manual screening of reference lists of relevant systematic reviews and meta-analyses. The overall search and selection process is summarized in Figure 1.

### 2.2. Study Selection

Studies were selected based on their relevance to the review objectives. Priority was given to randomized controlled trials, systematic reviews and meta-analyses, feasibility and pilot studies, prospective cohort studies, and clinical practice guidelines. Studies were considered eligible if they examined telehealth applications for shoulder assessment, rehabilitation, or integrated care delivery in adult populations, and reported clinical outcomes, diagnostic accuracy, patient satisfaction, adherence, or implementation factors. Studies were excluded if they were conference abstracts, editorials, or case reports, or if they addressed telehealth exclusively in non-shoulder musculoskeletal conditions without shoulder-specific data. Following the full screening process, a total of 97 articles were included in the final narrative synthesis.

### 2.3. Evidence Appraisal

No formal data extraction protocol or risk-of-bias assessment was applied, consistent with the narrative review methodology. The certainty of evidence is discussed qualitatively throughout the manuscript, drawing on GRADE assessments reported in included systematic reviews where available. For primary studies not covered by existing syntheses, methodological limitations are noted within the relevant sections of the review.

## 3. Tele-Diagnostics in Shoulder Disorders

The following two sections (Section 3 and Section 4) collectively constitute the results of this narrative review, presenting the synthesized evidence organized thematically by domain of telehealth application. The virtual evaluation of shoulder pathology represents a significant paradigm shift in musculoskeletal assessment, requiring adaptation of traditional physical examination techniques to remote delivery formats. Telemedicine-based shoulder examinations typically employ synchronous video consultations, where clinicians guide patients through self-performed or proxy-assisted examination maneuvers in real time [20]. The shoulder, as a complex anatomical region comprising four articulations with the widest range of motion in the human body, presents unique challenges for remote assessment [21,22]. Nevertheless, emerging evidence suggests that with appropriate protocols and patient guidance, clinically useful data may be obtained through telehealth platforms in selected presentations.

Comparative studies evaluating the diagnostic accuracy of telehealth shoulder examinations against traditional in-person assessments have demonstrated promising results. Key studies validating tele-diagnostic approaches in shoulder disorders are summarized in Table 1.

Bradley and colleagues conducted a pivotal study comparing physician-guided, patient-performed standardized telehealth examinations (STEs) with standard clinical examinations (SCEs) for detecting rotator cuff tears, using magnetic resonance imaging as the reference standard [16]. Among 62 consecutive patients with shoulder pain, no significant difference was observed in overall diagnostic effectiveness between the two assessment platforms (*p* = 0.98), providing initial evidence of feasibility and suggesting potential non-inferiority of telehealth evaluation in this context. Similarly, Wang et al. examined the reliability of video-guided telemedicine examinations across 40 physical examination maneuvers and reported overall agreement of 76.4% between in-person and telemedicine assessments, with range-of-motion evaluations demonstrating the highest reliability (KR-20 = 0.700) [23].

The reliability of specific examination components varies considerably in the telehealth setting. Range-of-motion assessments consistently demonstrate the highest reproducibility, with telemedicine maneuvers examining ROM limitations achieving sensitivity of 66.5%, specificity of 81.0%, and likelihood ratios of 6.06 for detecting positive findings [16,23]. Conversely, evaluations of glenohumeral instability present the greatest challenge, with apprehension testing showing the lowest reliability (KR-20 = 0.170) and sensitivity (23.5%) in remote formats [16]. Rabin et al. demonstrated high agreement regarding diagnosis (κ = 0.78) and moderate agreement for intervention selection (κ = 0.48) between video and face-to-face examinations, suggesting that telehealth assessment may inform clinical decision-making for selected shoulder pathologies, though generalizability remains uncertain [12].

The development of standardized telehealth examination protocols has been instrumental in optimizing remote shoulder assessment. The Bari Shoulder Telemedicine Examination Protocol (B-STEP), developed through consensus among ten orthopedic surgeons, provides a systematic framework for remote shoulder evaluation encompassing history, inspection, palpation guidance, range-of-motion assessment, and specific clinical tests [12]. This protocol demonstrated completion of 100% of American Shoulder and Elbow Surgeons (ASES) score items and at least 87.5% of Constant score components via webcam examination. Similarly, the Hospital for Special Surgery virtual shoulder examination protocol offers comprehensive guidance for clinicians, including verbal instructions and annotated demonstration images for patient-performed maneuvers [25]. These standardized approaches facilitate consistency across practitioners and optimize information yield from virtual encounters.

Technological advances in digital measurement tools have expanded the precision of remote shoulder assessment. Smartphone applications utilizing built-in accelerometers and gyroscopes have demonstrated validity and reliability for measuring active shoulder range of motion in standing positions, with results comparable to universal goniometer measurements [26]. Furthermore, wearable inertial measurement unit (IMU) systems have been validated against optical motion capture for shoulder kinematics in clinical contexts, offering potential for objective, quantifiable assessments [34]. These technologies may contribute to more standardized measurements, though clinical validation in routine telehealth settings remains limited, and facilitate remote monitoring of rehabilitation progress [26,34].

The integration of artificial intelligence and machine learning into shoulder tele-diagnostics represents an emerging frontier with considerable potential. Deep learning algorithms have shown promising results for automated detection of rotator cuff tears in research settings, though performance in routine clinical practice has not been established [35]. Research in AI applications for shoulder surgery has increased exponentially, with publications growing six-fold between 2018 and 2021 [29]. Algorithmic assessment of shoulder function using smartphone video capture and machine learning offers potential for automated, objective evaluation of movement patterns in remote settings [30]. However, systematic reviews caution that current AI applications in shoulder surgery require further validation, with many models lacking external validation and demonstrating variable performance metrics [36]. Critical appraisal of the AI and machine learning literature in shoulder care reveals several important methodological limitations that temper enthusiasm for these technologies. The majority of published diagnostic algorithms have been developed and validated on single-institution datasets, raising concerns about external validity and generalizability across different imaging protocols, patient populations, and clinical settings. Reported performance metrics such as AUC values frequently reflect internal validation only, with limited evidence of prospective external validation in independent cohorts [29]. Furthermore, calibration, the agreement between predicted probabilities and observed outcomes, is rarely reported, despite its clinical importance for decision support applications. The readiness of current AI tools for routine clinical implementation therefore remains limited; most algorithms described in this review should be considered at the proof-of-concept or early validation stage rather than as deployment-ready clinical tools [37]. Clinicians and healthcare systems considering adoption of AI-assisted shoulder diagnostics should await evidence from prospective, multicenter validation studies before integration into routine practice.

**Figure 2:**
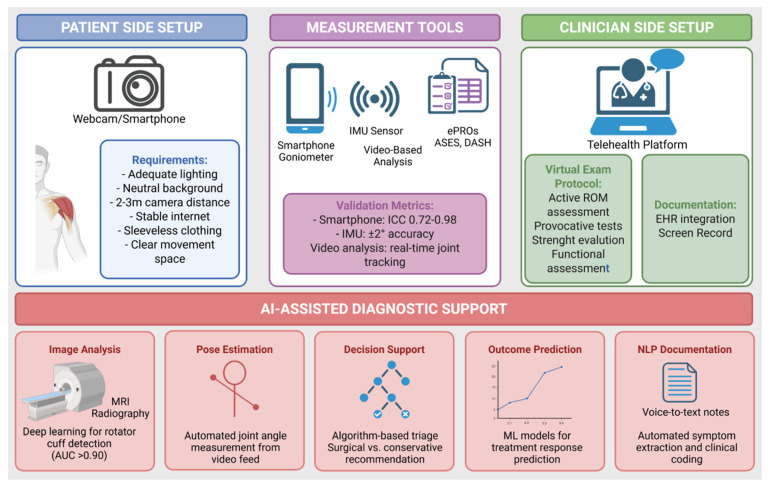
Tele-diagnostic tools and clinical setups. Overview of patient-side requirements, digital measurement tools, clinician interface, and AI-assisted diagnostic support for remote shoulder assessment. ePROs, electronic patient-reported outcomes; IMU, inertial measurement unit.

Patient satisfaction with telehealth shoulder consultations has been generally high across published studies, though reporting bias cannot be excluded. Systematic reviews of telemedicine in orthopedic surgery report that eleven of fifteen comparative studies demonstrated high patient satisfaction with virtual care, while nine showed equivalent clinical outcomes to traditional consultations [38]. Studies specifically examining shoulder and elbow telehealth visits during the COVID-19 pandemic revealed high satisfaction rates, with patients particularly valuing reduced travel time and infection risk [31,32]. Nevertheless, important limitations persist. The inability to perform hands-on palpation, passive-range-of-motion assessment, and provocative testing requiring examiner-applied resistance fundamentally constrains diagnostic precision. Conditions requiring tactile feedback, including subtle instability, specific impingement patterns, and neurovascular assessment, remain challenging to evaluate remotely. Patient factors including technological literacy, device availability, and physical capability to perform self-examination also influence diagnostic yield [18,21]. An overview of the technical and clinical components required for tele-diagnostic shoulder assessment, including patient-side setup, measurement tools, clinician interface, and AI-assisted diagnostic support, is summarized in Figure 2.

Collectively, the available evidence suggests that tele-diagnostic approaches for shoulder disorders demonstrate acceptable reliability for range-of-motion assessment and general diagnostic classification, with moderate agreement compared to in-person examination. However, the certainty of this evidence must be interpreted cautiously. Most validation studies are small, single-center, and conducted under controlled conditions that may not reflect routine clinical practice. Glenohumeral instability assessment remains consistently unreliable in remote formats across studies. The inability to perform palpation, passive motion testing, and resistance-based provocative maneuvers represents a fundamental and likely irreducible limitation of remote shoulder examination. Based on available evidence, tele-diagnostics may be considered a reasonable adjunct or triage tool for selected shoulder presentations, but cannot be regarded as equivalent to comprehensive in-person evaluation for all pathologies. The clearest clinical take-home message from the available tele-diagnostic evidence is therefore one of appropriate positioning: remote shoulder assessment is most valuable as a triage, monitoring, and follow-up tool for lower-complexity presentations, rather than as a first-line diagnostic instrument for conditions where tactile examination is diagnostically essential.

## 4. Tele-Rehabilitation in Shoulder Disorders

Tele-rehabilitation has emerged as a viable alternative to conventional face-to-face physiotherapy for patients with shoulder disorders, encompassing both postoperative and conservative management pathways [39]. The evidence base has expanded considerably in recent years, with randomized controlled trials investigating various technological modalities across diverse shoulder pathologies including rotator cuff tears, shoulder arthroplasty, subacromial pain syndrome, and adhesive capsulitis. The COVID-19 pandemic served as a catalyst for rapid adoption of remote rehabilitation services, generating substantial new evidence on feasibility, effectiveness, and patient acceptance while accelerating technological innovation in this field [39,40,41]. The evidence base has expanded considerably in recent years, with randomized controlled trials investigating various technological modalities across diverse shoulder pathologies including rotator cuff tears, shoulder arthroplasty, subacromial pain syndrome, and adhesive capsulitis; key trials are summarized in Table 2.

The postoperative rehabilitation of rotator cuff repair represents the most extensively studied application of tele-rehabilitation in shoulder disorders. Shim et al. compared augmented reality-based digital rehabilitation with conventional brochure-based home exercises in 115 patients following arthroscopic rotator cuff repair [17]. The digital healthcare group demonstrated greater improvement in the Simple Shoulder Test score at 12 weeks postoperatively (mean difference 6.24 ± 2.63 versus 5.04 ± 2.86; *p* = 0.025). Furthermore, significant group-by-time interactions were observed for the Shoulder Pain and Disability Index, Disabilities of the Arm, Shoulder and Hand, and EuroQol 5-Dimension 5-Level questionnaire scores over the 24-week follow-up period. Participant satisfaction with the augmented reality system was high, with an average score of 3.2 out of 4 across satisfaction domains, indicating strong patient acceptance of this technology [42]. Correia et al. similarly found comparable improvements in Constant–Murley scores between digitally assisted and conventional rehabilitation groups, with 96.9% of patients achieving clinically significant outcomes at one year [13].

Evidence for tele-rehabilitation following shoulder arthroplasty has emerged from recent trials. A 2025 randomized controlled trial by O’Reilly et al. comparing tele-rehabilitation versus in-person physical therapy in 81 patients undergoing anatomic total shoulder arthroplasty or reverse shoulder arthroplasty found no statistically significant differences in patient-reported outcome measures or range-of-motion outcomes at one year postoperatively [42]. Both cohorts followed similar patterns of improvement, suggesting that tele-rehabilitation may offer comparable outcomes while providing convenience, reducing travel requirements for patients, though longer follow-up data are lacking. Kane et al. demonstrated that telehealth platforms could safely deliver early postoperative care following rotator cuff repair, with no significant differences in American Shoulder and Elbow Surgeons scores or visual analog pain scales compared to in-person visits [43]. These findings suggest potential for integrating telehealth into postoperative follow-up protocols, pending confirmation in larger trials.

For conservative management of rotator cuff-related shoulder pain, the INTEL trial by Malliaras et al. provided foundational feasibility evidence for internet- and tele-rehabilitation-delivered care [9]. This pilot trial demonstrated excellent retention rates (94%) and acceptable adherence in the tele-rehabilitation group (92% completing two to three prescribed sessions per week) compared to internet-only delivery (67%), suggesting that synchronous tele-rehabilitation sessions with real-time clinician interaction may enhance exercise adherence compared to asynchronous approaches [9]. Pak et al. compared fully remote digital physical therapy using wearable motion sensors providing real-time biofeedback with conventional in-person therapy for chronic shoulder pain, finding clinically meaningful improvements in both groups without significant between-group differences [45]. The digital intervention utilized sensor-guided exercises with immediate feedback on movement quality, representing an advancement over simple video-based instruction.

Subacromial pain syndrome has been addressed through multiple tele-rehabilitation approaches. Çelik and Tuncer compared manual therapy with exercise to synchronized tele-rehabilitation with self-applied manual therapy in 60 patients, finding that both groups achieved comparable outcomes for pain, range of motion, and Quick-DASH scores [44]. This study demonstrated that patients could effectively perform self-manual therapy techniques when guided remotely. Pastora-Bernal and colleagues (2018) demonstrated that tele-rehabilitation following arthroscopic subacromial decompression achieved similar functional improvements to face-to-face physiotherapy (Constant–Murley score improvement from 43.50 to 68.50 points) while being more cost-effective [47]. For adhesive capsulitis, augmented reality-based systems utilizing motion sensors and real-time feedback have shown promise in enhancing exercise compliance [27,47].

Tele-rehabilitation modalities have evolved substantially, ranging from simple video-based interventions to sophisticated augmented reality systems with wearable sensors capable of tracking movement in real time. A systematic review and meta-analysis by Huang et al. synthesized evidence from seven randomized trials involving 508 participants with shoulder disorders, finding that tele-rehabilitation demonstrated superior improvements in range of motion across all measured planes, functional outcomes (SPADI and Quick-DASH scores), and quality of life compared to home-based exercise alone [40]. Pain relief was comparable between groups overall, with significant benefits observed when tele-rehabilitation continued for more than 12 weeks, suggesting a duration-dependent effect that underscores the importance of sustained remote monitoring and guidance [39].

Available evidence suggests that tele-rehabilitation may be non-inferior to standard in-person physiotherapy for most shoulder disorders. The relatively strongest evidence within this field supports postoperative rehabilitation following rotator cuff repair, where multiple randomized controlled trials have demonstrated comparable outcomes with high patient satisfaction [40,48]. For conservative management of rotator cuff-related shoulder pain, subacromial pain syndrome, and adhesive capsulitis, emerging evidence supports feasibility and preliminary effectiveness, though larger confirmatory trials are warranted. The choice of tele-rehabilitation modality should consider patient preferences, technological literacy, and specific rehabilitation goals for each clinical scenario [40,48].

The available randomized controlled trial evidence consistently demonstrates non-inferiority of tele-rehabilitation compared to conventional physiotherapy across multiple shoulder pathologies and surgical contexts. However, several methodological limitations constrain the certainty of these conclusions. First, the majority of trials follow participants for only 8 to 12 weeks, which is insufficient to evaluate long-term functional recovery, recurrence, or the durability of treatment effects in chronic shoulder conditions. Second, sample sizes across most trials are modest, limiting statistical power to detect clinically meaningful between-group differences. Third, blinding of participants and therapists is inherently impossible in rehabilitation trials, introducing performance and detection bias. Fourth, heterogeneity in intervention delivery, technological platforms, outcome measures, and patient populations limits cross-study comparability. Using GRADE terminology, the certainty of evidence supporting tele-rehabilitation for shoulder disorders is currently rated as low to moderate, consistent with recent systematic reviews and meta-analyses in this field. Larger, adequately powered trials with standardized outcome measures and longer follow-up durations are needed before definitive clinical recommendations can be established. Collectively, these findings suggest a pragmatic clinical message: tele-rehabilitation is ready for integration into routine shoulder care pathways for appropriately selected patients, with the strongest justification for postoperative rotator cuff rehabilitation, while remaining a complement rather than a universal replacement for in-person physiotherapy.

## 5. Patient Engagement, Adherence and Implementation Factors in Shoulder Tele-Rehabilitation

Exercise adherence constitutes a fundamental determinant of outcomes in shoulder rehabilitation [49], yet non-adherence to prescribed home exercise programs remains pervasive across musculoskeletal populations. Estimates suggest that 30–65% of patients with musculoskeletal conditions fail to complete their prescribed rehabilitation exercises, with consequences including prolonged disability, suboptimal recovery, and unnecessary escalation to surgical interventions [50]. In the specific context of shoulder pain, a 2024 study by Francisco et al. found that fewer than half of individuals with chronic shoulder pain adhered to an unsupervised home exercise booklet, with the most frequently cited barriers being pain during exercise, time constraints, and competing commitments; notably, enjoying the exercises was the strongest facilitator of adherence [51]. These findings underscore the need for innovative delivery models that address modifiable barriers and enhance the rehabilitative experience for patients with shoulder disorders.

### 5.1. Patient Adherence and Engagement

Participants across the included tele-rehabilitation trials were predominantly middle-aged to older adults, with mean ages generally ranging from 45 to 68 years depending on the clinical indication, with younger cohorts in conservative management studies and older cohorts in postoperative arthroplasty trials, a demographic profile that has direct implications for digital literacy, technology adoption, and long-term adherence. Tele-rehabilitation has emerged as a potentially promising strategy to improve exercise adherence in shoulder conditions by providing structured guidance, real-time feedback, and sustained clinician contact that are absent from traditional unsupervised home programs. A systematic review by Simmich et al. synthesizing evidence across musculoskeletal tele-rehabilitation trials found that real-time video-based tele-rehabilitation demonstrated comparable or superior attendance and adherence rates relative to in-person physiotherapy, though the certainty of this evidence remains low, with consistently high patient satisfaction [52]. In a randomized controlled trial comparing fully remote digital physical therapy to conventional in-person rehabilitation for chronic shoulder pain, Pak et al. reported high adherence and satisfaction in both groups, with no clinically meaningful differences in pain or functional outcomes, suggesting the potential viability of remote delivery as a scalable care model in selected population [45]. Similarly, Janela et al. demonstrated that an asynchronous, tailored digital rehabilitation program for chronic shoulder pain achieved clinically meaningful improvements in QuickDASH scores, pain, and mental health outcomes, with high patient engagement and satisfaction throughout the 12-week program [53].

Condition-specific evidence further supports the role of tele-rehabilitation in sustaining patient engagement across diverse shoulder pathologies. For rotator cuff-related conditions, the INTEL trial demonstrated that tele-rehabilitation combined with recommended care achieved superior exercise completion rates compared to internet-delivered exercise alone, highlighting the importance of clinician interaction in maintaining motivation [54]. In postoperative settings, Martinez-Rico et al. showed that a phone assistance nursing program significantly improved adherence to home exercises and functional outcomes in patients following shoulder instability surgery, suggesting that even low-technology remote contact can meaningfully enhance engagement [54]. Choi et al. found that smartphone application-supported self-rehabilitation for frozen shoulder achieved outcomes comparable to conventional physiotherapy, attributing the success partly to app-based exercise reminders and visual guidance that reinforced correct technique [55]. These studies collectively indicate that the continuous connection afforded by telehealth platforms addresses one of the most critical gaps in traditional shoulder rehabilitation: the loss of therapeutic contact between clinic visits.

### 5.2. Patient Satisfaction

Digital engagement strategies, including gamification, biofeedback, and augmented reality, represent evolving approaches to enhance adherence within shoulder tele-rehabilitation programs. Marley et al. conducted a multicenter randomized controlled trial comparing gamified rehabilitation using depth-sensor exergames with standard physiotherapy following arthroscopic shoulder surgery and found equivalent clinical outcomes, with the gamified group demonstrating sustained motivation throughout the rehabilitation period [46]. Wörner et al. reported that a digitally delivered exercise and education program produced significant improvements in pain and disability among patients with shoulder pain within three months, suggesting that structured digital content can effectively support behavioral change [56]. Motion-tracking digital platforms incorporating real-time biofeedback have demonstrated outcomes comparable to conventional rehabilitation following arthroscopic rotator cuff repair while reducing the number of required face-to-face sessions, thereby lowering patient burden and healthcare resource utilization [12].

Patient satisfaction with tele-rehabilitation and telehealth follow-up for shoulder disorders has been consistently high across multiple studies and clinical contexts. Markus et al. prospectively compared telemedicine and in-person follow-up visits after arthroscopic shoulder surgery in 96 patients and found no significant difference in overall care satisfaction scores between groups [57]. O’Donnell et al. demonstrated that virtual postoperative visits for shoulder surgery were 54% less costly and 88% shorter than in-person visits, with all telehealth patients reporting the experience as safe and convenient and expressing high satisfaction [58]. Sabbagh et al. similarly found that patients who underwent telemedicine follow-up for rotator cuff repair and total shoulder arthroplasty during the COVID-19 pandemic showed no difference in patient-reported outcome measures or satisfaction compared to traditional in-person follow-up, although the majority still expressed a preference for face-to-face visits when feasible [59]. Cha et al. reported comparable satisfaction scores between telemedicine and clinic follow-up visits among patients undergoing shoulder arthroscopy in a rural setting, noting that telehealth was particularly valued for reducing travel burden [60].

### 5.3. Clinician Perspectives

The perspective of clinicians regarding tele-rehabilitation for shoulder and musculoskeletal conditions is a critical implementation factor that influences adoption and sustainability. A qualitative focus group study by Sia et al. exploring physiotherapists’ perceptions of tele-rehabilitation for musculoskeletal disorders identified perceived benefits including time savings and cost reduction for patients, but also highlighted barriers such as the inability to perform manual therapy, difficulty assessing movement quality remotely, and concerns about patient safety during unsupervised exercises [61]. Baroni et al. noted that suboptimal tele-rehabilitation implementation may have occurred in countries where clinicians lacked prior training in digital health delivery, emphasizing the need for structured curricula in telehealth competencies [62]. A focus group study specifically examining physiotherapist perspectives on virtual reality-supported shoulder rehabilitation found that clinicians recognized the therapeutic potential of immersive technology but expressed concerns about the evidence base, patient suitability criteria, and the risk of reinforcing incorrect movement patterns without hands-on correction [63]. The American Physical Therapy Association’s 2024 clinical practice guideline acknowledged that research about the education and development of clinicians in tele-rehabilitation practice may address perceived or real barriers, and recommended shared decision-making between clinicians and patients regarding service delivery options [64].

### 5.4. Digital Divide and Health Equity

Equitable access to tele-rehabilitation services remains a significant implementation challenge, with disparities in digital readiness disproportionately affecting populations most likely to benefit from remote shoulder care. Falvey et al., using nationally representative data from the United States, found that only two-thirds of older rehabilitation users were considered ready to participate in video-based tele-rehabilitation, with significantly lower readiness rates among racial and ethnic minorities, financially strained individuals, and rural-dwelling older adults [65]. However, evidence suggests that the digital divide may be less prohibitive than assumed in practice: Areias et al. demonstrated that older adults enrolled in a digital musculoskeletal care program showed higher adherence, engagement, and satisfaction than younger counterparts, achieving clinically meaningful improvements in pain, mental health, and productivity [66]. These apparently contradictory findings suggest that once barriers to initial access are overcome, older adults may derive substantial benefit from digital shoulder rehabilitation programs. Addressing structural barriers such as broadband access, device availability, and digital literacy training is therefore essential for realizing the inclusive potential of tele-rehabilitation [65].

### 5.5. Cost-Effectiveness

**Figure 3:**
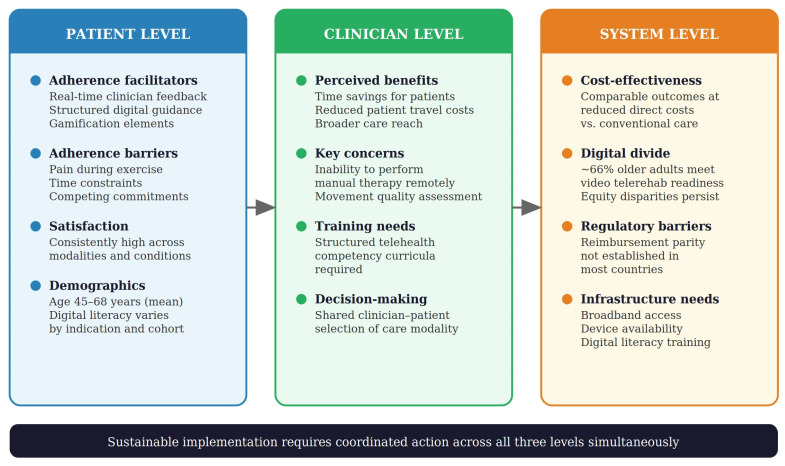
Multi-level determinants of patient engagement and implementation in shoulder tele-rehabilitation. Factors influencing adherence, adoption, and sustainability are organized across three interdependent levels: patient, clinician, and system. Sustainable implementation requires coordinated action across all three levels simultaneously.

Cost-effectiveness represents an important implementation consideration for healthcare systems contemplating the adoption of shoulder tele-rehabilitation. A systematic review and meta-analysis by Molina-Garcia et al. found that tele-rehabilitation for musculoskeletal disorders was cost-effective compared to conventional rehabilitation, with comparable clinical outcomes at reduced direct costs [67]. Türkmen et al. demonstrated that video-based rehabilitation for rotator cuff tears achieved similar functional improvements to face-to-face physiotherapy while reducing the number of clinic visits and associated costs [68]. In postoperative shoulder care, the shift toward hybrid models combining selective in-person visits with telehealth follow-up has been supported by evidence suggesting maintained clinical outcomes with improved resource allocation [28]. The clinical practice guideline from the American Physical Therapy Association further endorsed the integration of tele-rehabilitation into physical therapist practice, providing a regulatory framework that may facilitate broader institutional adoption [64]. The multi-level determinants of patient engagement and implementation in shoulder tele-rehabilitation are summarized in Figure 3.

Taken together, the evidence indicates that patient engagement and adherence in shoulder tele-rehabilitation are influenced by a complex interplay of patient-level, clinician-level, and system-level factors. At the patient level, the provision of structured digital exercise programs with real-time or asynchronous feedback, gamification elements, and regular clinician contact has consistently demonstrated favorable adherence outcomes compared to unsupervised exercise alone [46,51,53]. At the clinician level, attitudes toward telehealth are increasingly positive but remain tempered by concerns about diagnostic limitations and the loss of hands-on therapeutic skills [61,63]. At the system level, cost-effectiveness, regulatory acceptance, and strategies to bridge the digital divide are emerging as prerequisites for sustainable implementation [28,65,67].

## 6. Integration of Tele-Diagnostics and Tele-Rehabilitation

**Figure 4:**
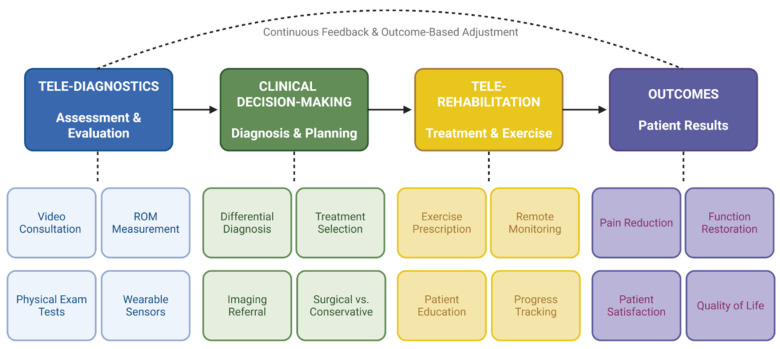
Conceptual framework for telehealth in shoulder disorders. Integrated pathway from tele-diagnostics through clinical decision-making and tele-rehabilitation to patient outcomes, with a continuous feedback loop enabling outcome-based treatment adjustments.

The convergence of tele-diagnostics and tele-rehabilitation represents a paradigm shift in the management of shoulder disorders, potentially enabling more continuous, data-driven care pathways that extend from initial assessment through complete functional recovery. Rather than operating as discrete services, diagnostic and therapeutic telehealth functions increasingly share common technological infrastructure, including wearable sensors, machine learning algorithms, and digital health platforms, creating integrated ecosystems in which clinical data may flow between assessment and treatment modules [69]. This integration aligns with the broader vision of personalized, predictive, participatory, precision, and preventive (P5) medicine applied to shoulder care, wherein continuous data capture informs iterative treatment refinement throughout the rehabilitation trajectory [69]. An integrated telehealth care pathway for shoulder disorders, linking tele-diagnostics, clinical decision-making, tele-rehabilitation, and patient outcomes with a continuous feedback loop, is illustrated in Figure 4.

Wearable sensor technology constitutes the primary hardware layer enabling diagnostic–therapeutic integration. A systematic review of remote monitoring devices for home-based shoulder rehabilitation identified wearable inertial measurement units (IMUs) as the most extensively validated monitoring modality, offering non-intrusive, continuous capture of kinematic data during both structured exercise sessions and daily functional activities [70]. These multi-axis sensor systems, which combine accelerometers, gyroscopes, and magnetometers, serve a dual purpose: during diagnostic assessment, they quantify range-of-motion deficits and movement quality; during rehabilitation, the same hardware provides real-time biofeedback and objective adherence tracking [71]. The potential significance of this convergence is that a single wearable device, deployed at the point of diagnosis, may transition into a rehabilitation monitoring tool, though this integration remains to be validated in large-scale clinical studies.

Machine learning algorithms represent the computational bridge between diagnostic data capture and therapeutic guidance within integrated platforms. Deep learning models trained on IMU sensor data from patients with adhesive capsulitis and rotator cuff disease have achieved test accuracies of 92.5% for classifying 11 distinct shoulder rehabilitation exercises, enabling automated monitoring of exercise quality and adherence without requiring synchronous clinician supervision [72]. Complementary work using Random Forest classifiers has demonstrated accuracies approaching 90% for exercise classification in both healthy subjects and patients with rotator cuff tears, following protocols developed by the American Society of Shoulder and Elbow Therapists [73]. Smartwatch-based systems have achieved classification accuracies exceeding 94% for temporal cross-validation and 88.9% for subject-stratified validation of shoulder physiotherapy exercises [74]. Critically, these same algorithms can simultaneously flag aberrant movement patterns, such as compensatory scapular elevation or reduced glenohumeral excursion, that carry diagnostic significance, potentially enabling clinicians to detect clinical deterioration or emerging complications during routine rehabilitation monitoring, though prospective clinical validation of this capability is still required.

Augmented reality (AR) platforms exemplify the practical realization of diagnostic–therapeutic integration in shoulder care. The UINCARE Home+ system, which provides AR-based visual guidance for exercise performance while simultaneously capturing kinematic data for remote clinician review, demonstrated significant functional improvements in a randomized controlled trial of 115 patients following rotator cuff repair [16]. The dual functionality of such platforms, wherein therapeutic instruction and diagnostic data collection occur in the same patient interaction, effectively extends diagnostic capability throughout the entire rehabilitation period, transforming each exercise session into both a treatment event and an assessment opportunity. This continuous diagnostic capacity addresses a fundamental limitation of traditional care models, in which diagnostic reassessment occurs only at scheduled follow-up appointments separated by weeks or months.

To illustrate how this integration operates in clinical practice, consider a patient undergoing postoperative rehabilitation following arthroscopic rotator cuff repair. At the initial remote assessment, a wearable IMU sensor is used to quantify baseline range-of-motion deficits and movement quality, establishing the diagnostic starting point for rehabilitation planning. The same device then transitions seamlessly into the rehabilitation phase, providing real-time biofeedback during home exercise sessions and continuously transmitting kinematic data to the supervising clinician. If the machine learning algorithm flags compensatory scapular elevation or reduced glenohumeral excursion during a routine exercise session, the clinician receives an automated alert and can adjust the rehabilitation protocol remotely without requiring an in-person visit. This single integrated workflow, encompassing remote diagnosis, supervised rehabilitation, continuous monitoring, and iterative clinical adjustment, illustrates the practical advantage of integrated telehealth platforms over sequential, separately delivered tele-diagnostic and tele-rehabilitation services [70,71,72,75].

The integration of home-based rehabilitation devices with telehealth monitoring has been directly evaluated in shoulder surgery populations. Greiner et al. reported outcomes from 132 patients who used a home-based Shoulder Strengthening and Stabilization System (SSS) combined with telehealth visits following shoulder surgery, finding that 96% reported positive impacts on rehabilitation, with significant reductions in both pain scores and analgesic use from the first to eighth postoperative telehealth session (*p* < 0.01), with 93% recommending the system upon protocol completion [75]. This model, which combines a physical rehabilitation device with scheduled remote clinical monitoring, demonstrates how integrated platforms can maintain therapeutic continuity while preserving clinician oversight across the postoperative period. Digital biofeedback platforms incorporating motion-tracking technology have similarly achieved outcomes comparable to conventional in-person rehabilitation following arthroscopic rotator cuff repair while reducing the number of required face-to-face sessions [12], and fully remote digital physical therapy has produced equivalent functional improvements for chronic shoulder pain [43], collectively supporting the feasibility of replacing sequential in-person diagnostic and therapeutic visits with continuous integrated remote care.

The translation of integrated telehealth systems into routine clinical practice is supported by evolving professional frameworks and post-pandemic utilization patterns. The American Physical Therapy Association’s 2024 clinical practice guideline on tele-rehabilitation provides an evidence-based framework for incorporating remote assessment alongside telehealth-delivered interventions, endorsing shared decision-making between clinicians and patients regarding the balance of in-person and virtual care components [64]. Post-pandemic analyses indicate sustained adoption of telehealth for shoulder follow-up, with evidence supporting hybrid models that combine selective in-person visits for procedures requiring hands-on assessment with telehealth for routine monitoring, exercise progression, and outcome tracking [28]. However, the optimal configuration of hybrid pathways, specifically which clinical decision points necessitate in-person diagnostic encounters and which can be safely managed through integrated remote assessment, remains to be defined through prospective comparative studies.

In summary, the integration of tele-diagnostics and tele-rehabilitation in shoulder disorders is enabled by three converging technological developments: wearable sensor hardware that captures clinically meaningful kinematic data during both assessment and exercise [69,76]; machine learning algorithms that simultaneously classify exercise performance and detect diagnostically significant movement abnormalities [72,73,74]; and digital health platforms that present this information to clinicians and patients through intuitive interfaces supporting real-time or asynchronous decision-making [16,75]. The central advantage of integration over parallel but separate diagnostic and therapeutic telehealth services is the creation of a continuous feedback loop (Figure 3) in which rehabilitation performance data inform ongoing diagnostic refinement, and diagnostic findings drive immediate therapeutic adjustment, a model that more closely approximates the iterative clinical reasoning of face-to-face care than either tele-diagnostics or tele-rehabilitation can achieve independently.

## 7. Limitations and Challenges of Tele-Rehabilitation and Tele-Diagnostics

Despite the promise of tele-rehabilitation, several significant limitations impede its widespread adoption for shoulder disorders. A fundamental barrier remains the inability to perform hands-on physical examination remotely. Palpation for tenderness localization, manual muscle testing against resistance, and assessment of joint instability are essential components of shoulder evaluation that cannot be replicated virtually [22]. Even with patient-assisted examination techniques using household objects for resistance, diagnostic accuracy for specific shoulder pathologies remains suboptimal compared to in-person assessment [77]. Remote visual estimation of shoulder range of motion demonstrates high interobserver reliability (ICC 0.74–0.93), yet measurement errors frequently exceed minimal clinically important differences, limiting its utility for detecting meaningful clinical changes [23].

The digital divide presents substantial equity concerns in tele-rehabilitation implementation. Only approximately 66% of older adults who use rehabilitation services in the United States meet readiness criteria for video-based tele-rehabilitation [65]. Significant disparities exist across demographic groups, with substantially lower readiness rates among Black (39%), Hispanic (49%), and Asian/Pacific Islander/Native American (34%) populations compared to White older adults [65]. A rapid review examining ethics and equity in tele-rehabilitation found that disparities in socioeconomic status, geographic location, and racial/ethnic backgrounds significantly impact tele-rehabilitation utilization, while ethical concerns regarding privacy, security, and patient autonomy remain inadequately addressed across most interventions [78]. Surveys of physiotherapists in developing countries reveal that technical issues (58.2%) and lack of awareness (59.9%) constitute the primary barriers to tele-rehabilitation implementation [79].

The current evidence base for shoulder tele-rehabilitation suffers from methodological limitations. Multiple recent meta-analyses report low-to-very-low certainty of evidence using GRADE criteria, primarily due to heterogeneous intervention protocols, small sample sizes, high risk of bias, and the inherent impossibility of blinding participants to treatment allocation [39,41]. The lack of standardized outcome measures and intervention descriptions across studies limits the ability to draw definitive conclusions or develop evidence-based clinical guidelines. A systematic review specifically examining tele-rehabilitation for shoulder pain found very-low-to-low-quality evidence supporting its use, with most studies following participants for only 8–12 weeks [80]. A state-of-the-art review of musculoskeletal tele-rehabilitation emphasizes that cost-effectiveness studies remain scarce, and implementation trials are particularly lacking in low- and middle-income countries where access barriers are most pronounced [62].

Tele-rehabilitation cannot replicate manual therapy techniques that may provide additive benefits for certain shoulder conditions. While a recent randomized controlled trial found no significant differences between synchronized tele-rehabilitation with self-manual therapy and in-person manual therapy for subacromial pain syndrome, the study acknowledges that tele-rehabilitation remains inherently limited in delivering specialized hands-on interventions [45]. Regarding exercise adherence, a systematic review found that real-time video tele-rehabilitation shows potentially favorable effects, with 8% higher attendance and 9% higher adherence compared to in-person physiotherapy, though the certainty of evidence remains low [51]. Privacy and data security represent persistent concerns, as many commercial videoconferencing platforms lack adequate encryption and no universal standards exist for protecting sensitive health information transmitted during virtual sessions [78]. The APTA Clinical Practice Guideline on tele-rehabilitation emphasizes that clinicians must carefully assess patient suitability and address technological barriers while maintaining appropriate documentation and safety standards [64]. Finally, most published trials demonstrate short-term effectiveness (8–12 weeks), with substantial gaps in long-term outcome data needed to inform chronic condition management and determine whether tele-rehabilitation benefits persist beyond the intervention period [39,67].

Regulatory, reimbursement, and medico-legal barriers represent additional implementation challenges that remain incompletely resolved across most healthcare systems. Telehealth regulations vary substantially between jurisdictions, with significant differences in licensure requirements for cross-border practice and the regulatory status of AI-assisted diagnostic tools creating uneven access to telehealth shoulder care [81]. Reimbursement parity between telehealth and in-person physiotherapy has not been established in many countries, creating financial disincentives for providers and patients that constrain adoption independently of clinical evidence [82]. Medico-legal questions regarding liability for adverse events during unsupervised home exercise, diagnostic errors arising from remote assessment limitations, and standards for informed consent in telehealth practice remain insufficiently standardized in orthopedic and rehabilitation contexts [83]. The present review has several inherent limitations that should be acknowledged. As a narrative review, the study selection process relied on structured but non-systematic searching, and the inclusion of studies was not exhaustive; some relevant publications may therefore have been missed. The restriction to English-language publications may have introduced language bias. Additionally, the heterogeneity of the included studies, varying in design, population, intervention type, and outcome measures, limits the strength of conclusions that can be drawn across domains. These limitations are balanced by the breadth of coverage achieved across both tele-diagnostic and tele-rehabilitative evidence, the explicit use of SANRA guidelines to ensure methodological transparency, and the novelty of examining both telehealth domains within a single integrative synthesis specifically focused on shoulder disorders.

## 8. Future Directions

Future research in tele-rehabilitation and tele-diagnostics for shoulder disorders should prioritize strategies for improving long-term patient adherence, which remains one of the most critical and incompletely resolved challenges in remote care delivery. AI and machine learning systems show particular promise in this regard, with deep learning algorithms enabling automated classification of rehabilitation exercises and real-time movement feedback that may sustain motivation during unsupervised home exercise [72]. Wearable inertial measurement units and novel strain sensors further support adherence by providing continuous progress monitoring and fatigue estimation during home-based shoulder rehabilitation [27,84,85].

Virtual and augmented reality platforms offer an adherence-enhancing dimension through immersive and gamified exercise environments, with early trials demonstrating acceptable clinical performance and patient engagement in post-arthroscopic and rotator cuff repair contexts [12,16,76,86].

Smartphone-based pose estimation and clinometer applications represent the most scalable avenue for adherence monitoring, enabling automated movement quality assessment and timely clinician intervention when technique or participation deteriorates [33,34,87,88].

Digital twin technology represents an emerging frontier, with patient-specific computational models integrating biomechanical, imaging, and wearable sensor data to simulate individualized rehabilitation scenarios and predict treatment outcomes [89]. These virtual replicas enable clinicians to optimize rehabilitation protocols and anticipate complications before they occur. Hybrid care models combining tele-rehabilitation with periodic in-person sessions are demonstrating effectiveness comparable to traditional rehabilitation, with randomized trials showing that digitally assisted rehabilitation can achieve superior outcomes compared to conventional home exercise programs [12,43].

## 9. Conclusions

This narrative review synthesizes current evidence on tele-diagnostics and tele-rehabilitation in shoulder disorders. Three principal evidence-based messages emerge: tele-diagnostics is best positioned as a triage and monitoring adjunct rather than a replacement for in-person examination; tele-rehabilitation is non-inferior to conventional physiotherapy for appropriately selected patients, with the strongest evidence supporting postoperative rotator cuff rehabilitation; and hybrid care models combining selective in-person contact with remote delivery represent the most pragmatic and evidence-supported pathway for implementation. However, the certainty of available evidence is currently low to moderate, primarily due to short follow-up durations, modest sample sizes, and heterogeneous intervention protocols. Implementation remains challenged by the digital divide, variable clinician readiness, and evolving reimbursement frameworks. Emerging technologies including wearable sensors, machine learning, and augmented reality show considerable promise for integrated diagnostic–therapeutic platforms, but most remain at the proof-of-concept stage pending prospective multicenter validation.

Realizing this potential will require standardized outcome measures, longer-term trials, and targeted strategies to address health equity barriers.

## Figures and Tables

**Table 1:** Summary of key tele-diagnostic approaches and validation studies in shoulder disorders.

Author	Year	Condition	Method	Reliability/Validity
Group 1: Clinical Examination Validation				
Bradley et al. [16]	2021	Shoulder disorders (general)	Video consultation vs. in-person examination (RCT, *n* = 62)	Non-inferiority confirmed (*p* = 0.98); MRI as reference standard
Wang et al. [23]	2022	Shoulder pathology	Virtual physical examination with standardized maneuvers	Overall agreement 76.4%; KR-20 = 0.391; ROM highest reliability (KR-20 = 0.700)
Rabin et al. [12]	2022	Shoulder disorders	Smartphone-based remote assessment	Diagnosis agreement κ = 0.78; intervention agreement κ = 0.48
Hwang et al. [24]	2023	Shoulder ROM	Telehealth ROM assessment	High interobserver reliability for active ROM measurements
Moretti et al. [25]	2023	Shoulder disorders	B-STEP protocol (Bologna Shoulder Tele-Examination Protocol)	100% ASES completion rate; 87.5% Constant score feasibility
Lamplot et al. [26]	2020	Shoulder examination	HSS virtual shoulder physical examination protocol	Standardized protocol with demonstration images validated
Group 2: Digital Measurement Tools				
Yeo et al. [27]	2021	Shoulder ROM	Smartphone goniometer application	ICC 0.72–0.98 compared to standard goniometry
Kim et al. [28]	2022	Shoulder ROM	Wearable IMU sensor system	±2° accuracy; 9-axis motion tracking validated
Group 3: AI/ML and Diagnostic Technology				
Lin et al. [29]	2023	Rotator cuff tears	Deep learning MRI analysis	AUC > 0.90 for tear detection
Familiari et al. [30]	2022	Rotator cuff pathology	Systematic review of AI/ML applications	6-fold publication increase 2018–2021; high diagnostic accuracy reported
Group 4: Telehealth Implementation and Patient Satisfaction				
Fahey et al. [31]	2022	Orthopedic conditions (systematic review)	Telemedicine consultations	11/15 studies: high patient satisfaction; 9/15: equivalent clinical outcomes
Ben-Ari et al. [32]	2021	Shoulder surgery patients	Postoperative telehealth follow-up	>76% patient satisfaction; comparable outcomes to in-person
Sahu et al. [33]	2022	Shoulder/elbow disorders	COVID-19 telehealth implementation	High patient acceptance; effective for routine follow-up

ASES, American Shoulder and Elbow Surgeons score; ICC, intraclass correlation coefficient; IMU, inertial measurement unit; KR-20, Kuder–Richardson Formula 20 reliability coefficient (range 0–1; higher values indicate greater reliability); MRI, magnetic resonance imaging; ROM, range of motion; RCT, randomized controlled trial; κ, Cohen’s kappa coefficient; AUC, area under the receiver operating characteristic curve; AI/ML, artificial intelligence/machine learning; B-STEP, Bologna Shoulder Tele-Examination Protocol; HSS, Hospital for Special Surgery.

**Table 2:** Summary of key randomized controlled trials on tele-rehabilitation in shoulder disorders. ASES, American Shoulder and Elbow Surgeons score; AR, augmented reality; PROMs, patient-reported outcome measures; PT, physical therapy; ROM, range of motion; RCT, randomized controlled trial; SPADI, Shoulder Pain and Disability Index; SST, Simple Shoulder Test; VAS, visual analog scale; Quick-DASH, Quick Disabilities of the Arm, Shoulder and Hand; NR, not reported.

Study	Design	Sample	Condition	Intervention	Follow-Up	Primary Outcome	Key Finding	Limitations
Shim et al. [17]	RCT	n = 115	Rotator cuff repair	AR-based digital rehabilitation vs. brochure-based home exercise	24 weeks	SST, SPADI, DASH	Superior SST improvement in digital group at 12 weeks	Single center; no blinding possible
O’Reilly et al. [42]	RCT	n = 81	Shoulder arthroplasty	Tele-rehabilitation vs. in-person PT	12 months	PROMs, ROM	No significant difference between groups	Heterogeneous surgical procedures
Kane et al. [43]	RCT	n = NR	Rotator cuff repair	Telehealth postoperative visits vs. in-person	12 weeks	ASES, VAS pain	No significant differences	Short follow-up; limited to postoperative visits only
Çelik & Tuncer [44]	RCT	n = 60	Subacromial pain syndrome	Tele-rehabilitation with self-manual therapy vs. in-person manual therapy	8 weeks	Pain, ROM, Quick-DASH	Comparable outcomes between groups	Short follow-up; self-manual therapy may not replicate in-person technique
Pak et al. [45]	RCT	n = NR	Chronic shoulder pain	Digital PT with wearable sensors vs. conventional PT	12 weeks	Pain, function	Clinically meaningful improvements in both groups; no between-group difference	Industry-funded study
Marley et al. [46]	RCT	n = NR	Post-arthroscopic surgery	Gamified rehabilitation vs. standard physiotherapy	NR	Clinical outcomes, motivation	Equivalent outcomes; sustained motivation in gamified group	Multicenter heterogeneity
Malliaras et al. [9]	RCT pilot	n = NR	Rotator cuff-related pain	Tele-rehabilitation + recommended care vs. internet-only	12 weeks	Retention, adherence	94% retention; superior adherence in tele-rehabilitation group	Pilot study; underpowered for efficacy

## Data Availability

The data supporting the findings of this study are available within the article.

## Abbreviations

The following abbreviations are used in this manuscript:

AI	Artificial Intelligence
APTA	American Physical Therapy Association
AR	Augmented Reality
ASES	American Shoulder and Elbow Surgeons
AUC	Area Under the Curve
B-STEP	Bari Shoulder Telemedicine Examination Protocol
COVID-19	Coronavirus Disease 2019
DASH	Disabilities of the Arm, Shoulder and Hand
ePROs	Electronic Patient-Reported Outcomes
GRADE	Grading of Recommendations, Assessment, Development and Evaluations
HSS	Hospital for Special Surgery
ICC	Intraclass Correlation Coefficient
IMU	Inertial Measurement Unit
KR-20	Kuder–Richardson Formula 20
ML	Machine Learning
MRI	Magnetic Resonance Imaging
P5	Personalized, Predictive, Participatory, Precision, and Preventive
Quick-DASH	Quick Disabilities of the Arm, Shoulder and Hand
RCT	Randomized Controlled Trial
ROM	Range of Motion
SCE	Standard Clinical Examination
SPADI	Shoulder Pain and Disability Index
SSS	Shoulder Strengthening and Stabilization System
STE	Standardized Telehealth Examination
VR	Virtual Reality
